# Supplementary material for: Allosteric regulation and crystallographic fragment screening of SARS-CoV-2 NSP15 endoribonuclease
Source: Nucleic Acids Res. 2023 Apr 28;51(10):5255–70. doi: 10.1093/nar/gkad314 (PMC10250223; doi:10.1093/nar/gkad314)
Supplement: gkad314_Supplemental_Files [file gkad314_supplemental_files.zip › Supplementary datasets.zip!/160/Refine_8/01-BUSTER/Cycle-4/shell.01/R_Rfree.html]

# Rxpct factors in resolution bins at cycle 0


|  |  |  |  |  |  |  |  |  |
| --- | --- | --- | --- | --- | --- | --- | --- | --- |
|  | **<d> (Å)**  Rxpct\_Work | Rxpct\_Free | Rxpct\_All | |  |  |  |  |
|  | 14.6046 | 0.3905 | 0.5812 | 0.3972 |  |
|  | 8.4320 | 0.2479 | 0.2768 | 0.2496 |  |
|  | 6.5314 | 0.2136 | 0.2615 | 0.2162 |  |
|  | 5.5200 | 0.1819 | 0.2358 | 0.1841 |  |
|  | 4.8682 | 0.1478 | 0.2032 | 0.1509 |  |
|  | 4.4035 | 0.1489 | 0.1858 | 0.1510 |  |
|  | 4.0506 | 0.1535 | 0.1848 | 0.1549 |  |
|  | 3.7709 | 0.1608 | 0.2412 | 0.1650 |  |
|  | 3.5421 | 0.1671 | 0.2031 | 0.1689 |  |
|  | 3.3505 | 0.1748 | 0.2447 | 0.1786 |  |
|  | 3.1870 | 0.1796 | 0.2359 | 0.1827 |  |
|  | 3.0453 | 0.1826 | 0.2271 | 0.1846 |  |
|  | 2.9209 | 0.1927 | 0.2127 | 0.1936 |  |
|  | 2.8107 | 0.1878 | 0.2395 | 0.1902 |  |
|  | 2.7120 | 0.1896 | 0.2192 | 0.1912 |  |
|  | 2.6231 | 0.1981 | 0.2178 | 0.1992 |  |
|  | 2.5423 | 0.2099 | 0.2205 | 0.2105 |  |
|  | 2.4686 | 0.2109 | 0.2225 | 0.2115 |  |
|  | 2.4010 | 0.2228 | 0.2320 | 0.2232 |  |
|  | 2.3386 | 0.2515 | 0.2674 | 0.2522 |  |
|  | **Overall** | 0.1891 | 0.2342 | 0.1914 |  |

# Rxpct factors in resolution bins at cycle 1


|  |  |  |  |  |  |  |  |  |
| --- | --- | --- | --- | --- | --- | --- | --- | --- |
|  | **<d> (Å)**  Rxpct\_Work | Rxpct\_Free | Rxpct\_All | |  |  |  |  |
|  | 14.6046 | 0.3906 | 0.5814 | 0.3973 |  |
|  | 8.4320 | 0.2480 | 0.2769 | 0.2497 |  |
|  | 6.5314 | 0.2137 | 0.2615 | 0.2163 |  |
|  | 5.5200 | 0.1820 | 0.2359 | 0.1842 |  |
|  | 4.8682 | 0.1478 | 0.2033 | 0.1509 |  |
|  | 4.4035 | 0.1490 | 0.1858 | 0.1510 |  |
|  | 4.0506 | 0.1536 | 0.1848 | 0.1550 |  |
|  | 3.7709 | 0.1607 | 0.2412 | 0.1650 |  |
|  | 3.5421 | 0.1670 | 0.2031 | 0.1688 |  |
|  | 3.3505 | 0.1747 | 0.2446 | 0.1785 |  |
|  | 3.1870 | 0.1796 | 0.2358 | 0.1827 |  |
|  | 3.0453 | 0.1826 | 0.2271 | 0.1846 |  |
|  | 2.9209 | 0.1927 | 0.2127 | 0.1936 |  |
|  | 2.8107 | 0.1878 | 0.2397 | 0.1902 |  |
|  | 2.7120 | 0.1896 | 0.2191 | 0.1912 |  |
|  | 2.6231 | 0.1980 | 0.2178 | 0.1992 |  |
|  | 2.5423 | 0.2098 | 0.2202 | 0.2104 |  |
|  | 2.4686 | 0.2108 | 0.2224 | 0.2114 |  |
|  | 2.4010 | 0.2228 | 0.2319 | 0.2232 |  |
|  | 2.3386 | 0.2515 | 0.2674 | 0.2522 |  |
|  | **Overall** | 0.1891 | 0.2341 | 0.1914 |  |

# Rxpct factors in resolution bins at cycle 2


|  |  |  |  |  |  |  |  |  |
| --- | --- | --- | --- | --- | --- | --- | --- | --- |
|  | **<d> (Å)**  Rxpct\_Work | Rxpct\_Free | Rxpct\_All | |  |  |  |  |
|  | 14.6046 | 0.3908 | 0.5816 | 0.3975 |  |
|  | 8.4320 | 0.2482 | 0.2769 | 0.2498 |  |
|  | 6.5314 | 0.2138 | 0.2615 | 0.2164 |  |
|  | 5.5200 | 0.1821 | 0.2360 | 0.1843 |  |
|  | 4.8682 | 0.1479 | 0.2034 | 0.1509 |  |
|  | 4.4035 | 0.1490 | 0.1859 | 0.1511 |  |
|  | 4.0506 | 0.1535 | 0.1847 | 0.1549 |  |
|  | 3.7709 | 0.1606 | 0.2412 | 0.1649 |  |
|  | 3.5421 | 0.1669 | 0.2031 | 0.1687 |  |
|  | 3.3505 | 0.1746 | 0.2444 | 0.1784 |  |
|  | 3.1870 | 0.1795 | 0.2356 | 0.1826 |  |
|  | 3.0453 | 0.1826 | 0.2273 | 0.1846 |  |
|  | 2.9209 | 0.1927 | 0.2128 | 0.1936 |  |
|  | 2.8107 | 0.1878 | 0.2399 | 0.1902 |  |
|  | 2.7120 | 0.1897 | 0.2191 | 0.1912 |  |
|  | 2.6231 | 0.1979 | 0.2180 | 0.1991 |  |
|  | 2.5423 | 0.2098 | 0.2201 | 0.2103 |  |
|  | 2.4686 | 0.2107 | 0.2222 | 0.2113 |  |
|  | 2.4010 | 0.2227 | 0.2318 | 0.2231 |  |
|  | 2.3386 | 0.2515 | 0.2672 | 0.2522 |  |
|  | **Overall** | 0.1891 | 0.2341 | 0.1914 |  |

# Rxpct factors in resolution bins at cycle 3


|  |  |  |  |  |  |  |  |  |
| --- | --- | --- | --- | --- | --- | --- | --- | --- |
|  | **<d> (Å)**  Rxpct\_Work | Rxpct\_Free | Rxpct\_All | |  |  |  |  |
|  | 14.6046 | 0.3891 | 0.5796 | 0.3958 |  |
|  | 8.4320 | 0.2477 | 0.2775 | 0.2495 |  |
|  | 6.5314 | 0.2151 | 0.2541 | 0.2173 |  |
|  | 5.5200 | 0.1833 | 0.2433 | 0.1858 |  |
|  | 4.8682 | 0.1479 | 0.2066 | 0.1512 |  |
|  | 4.4035 | 0.1497 | 0.1888 | 0.1519 |  |
|  | 4.0506 | 0.1528 | 0.1885 | 0.1544 |  |
|  | 3.7709 | 0.1589 | 0.2393 | 0.1632 |  |
|  | 3.5421 | 0.1648 | 0.2015 | 0.1667 |  |
|  | 3.3505 | 0.1723 | 0.2407 | 0.1760 |  |
|  | 3.1870 | 0.1784 | 0.2346 | 0.1815 |  |
|  | 3.0453 | 0.1821 | 0.2295 | 0.1842 |  |
|  | 2.9209 | 0.1916 | 0.2178 | 0.1928 |  |
|  | 2.8107 | 0.1872 | 0.2438 | 0.1898 |  |
|  | 2.7120 | 0.1902 | 0.2161 | 0.1916 |  |
|  | 2.6231 | 0.1984 | 0.2190 | 0.1996 |  |
|  | 2.5423 | 0.2107 | 0.2184 | 0.2111 |  |
|  | 2.4686 | 0.2111 | 0.2220 | 0.2117 |  |
|  | 2.4010 | 0.2223 | 0.2304 | 0.2226 |  |
|  | 2.3386 | 0.2524 | 0.2706 | 0.2532 |  |
|  | **Overall** | 0.1886 | 0.2346 | 0.1910 |  |
